# Age-Related Changes in the Expression of the Circadian Clock Protein PERIOD in *Drosophila* Glial Cells

**DOI:** 10.3389/fphys.2017.01131

**Published:** 2018-01-09

**Authors:** Dani M. Long, Jadwiga M. Giebultowicz

**Affiliations:** Department of Integrative Biology, Oregon State University, Corvallis, OR, United States

**Keywords:** glia, circadian biology, aging, *Drosophila*, glial clocks

## Abstract

Circadian clocks consist of molecular negative feedback loops that coordinate physiological, neurological, and behavioral variables into “circa” 24-h rhythms. Rhythms in behavioral and other circadian outputs tend to weaken during aging, as evident in progressive disruptions of sleep-wake cycles in aging organisms. However, less is known about the molecular changes in the expression of clock genes and proteins that may lead to the weakening of circadian outputs. Western blot studies have demonstrated that the expression of the core clock protein PERIOD (PER) declines in the heads of aged *Drosophila melanogaster* flies. This age-related decline in PER does not occur in the central pacemaker neurons but has been demonstrated so far in retinal photoreceptors. Besides photoreceptors, clock proteins are also expressed in fly glia, which play important roles in neuronal homeostasis and are further categorized into subtypes based on morphology and function. While previous studies of mammalian glial cells have demonstrated the presence of functional clocks in astrocytes and microglia, it is not known which glial cell types in *Drosophila* express clock proteins and how their expression may change in aged individuals. Here, we conducted immunocytochemistry experiments to identify which glial subtypes express PER protein suggestive of functional circadian clocks. Glial cell subtypes that showed night-time accumulation and day-time absence in PER consistent with oscillations reported in the pacemaker neurons were selected to compare the level of PER protein between young and old flies. Our data demonstrate that some glial subtypes show rhythmic PER expression and the relative PER levels become dampened with advanced age. Identification of glial cell types that display age-related dampening of PER levels may help to understand the cellular changes that contribute to the loss of homeostasis in the aging brain.

## Introduction

Many molecular, cellular, and physiological processes in most organisms are coordinated with the predictable changes of the 24-h solar day. The circadian clock provides the mechanism of time keeping that is based on a negative feedback loop of transcriptional activators and repressors that generate endogenous molecular oscillations of circa 24 h (Hardin, 2011). A core repressor in the clock mechanism is encoded by the gene *period* (*per*) and the translocation of the PER protein into the cell nuclei followed by its degradation is the fundamental feature of clock function (Hardin, 2011). In both mammals and *Drosophila*, the circadian system consists of central and peripheral clocks (Hardin and Panda, 2013). Central pacemaker neurons in mammals are located in the suprachiasmatic nuclei (SCN). The central pacemaker neurons driving behavioral rest/activity rhythms consist of a network of about 150 neurons in the *Drosophila* brain. In addition to these central pacemaker neurons, mammals have intrinsic peripheral clocks in cells of fat tissues, kidneys, liver, and most other organs. Many of these peripheral tissues that express autonomous oscillators coordinate local tissue-specific processes. Similarly to mammals, peripheral clocks are widespread in fly tissues and function independently of the central pacemaker coordinating tissue-specific physiological processes (Giebultowicz, 2001). Within the nervous system, peripheral clocks are present in retinal photoreceptor cells and in other sensory neurons. In addition to neurons, some glial cells rhythmically express circadian clock genes in both mammals (Prolo et al., 2005; Marpegan et al., 2011; Hayashi et al., 2013) and in *Drosophila* (Ng et al., 2011). Early studies showed that PER protein is expressed in non-neuronal cells (Zerr et al., 1990; Ewer et al., 1992) and suggested that PER expression in these presumed glia is sufficient for manifestation of behavioral rhythmicity (Ewer et al., 1992). However, which glial cell subtypes express the PER-based oscillator and what their roles may be in the timekeeping processes remain poorly understood.

As organisms age, circadian rhythms tend to dampen as demonstrated in behavioral and molecular experiments both in mammals (Reddy and O'Neill, 2010) and *Drosophila* (Giebultowicz and Long, 2015). This phenomenon is implicated in declining cellular homeostasis and in various pathologies of aging, including altered inflammatory responses (Fonken et al., 2016), neurodegenerative diseases (Musiek et al., 2013) and impaired memory formation (Kondratova et al., 2010). In addition, physiological aging and late life diseases are accelerated by chronic disruption of clock functions in mammals (Kondratov et al., 2006; Antoch et al., 2008; Yu and Weaver, 2011; Hastings and Goedert, 2013). Similar to mammals, mutations in core clock genes accelerate aging phenotypes in *Drosophila* (Krishnan et al., 2009). Disruptions of the circadian clock in flies predispose them to neurodegeneration, although it is not known which clocks are involved (Krishnan et al., 2009, 2012). It was shown that *Drosophila per*^*01*^ mutants have increased levels of oxidative damage and neurodegeneration compared to age-matched controls (Krishnan et al., 2009). However, it is not known to what extent *per* mRNA is expressed in the glia, and consequently, whether loss of *per* in these cells could contribute to neurodegeneration and aging in general.

Glial cells play important roles in such processes as neuronal guidance during development, neuronal homeostasis, clearance of damaged tissues, and neurotransmitter recycling (Freeman and Doherty, 2006; Edwards and Meinertzhagen, 2010; Stork et al., 2012). Recent studies implicate mammalian astrocytes in neuroprotection via involvement in toxin clearance from the brain during sleep (Xie et al., 2013) and removal of damaged mitochondria from neurons in the process of transmitophagy (Davis et al., 2014). Glial cells were first classified based upon their location within the brain as surface, cortex, and neuropil glia. Recent classifications in mammals include astrocytes, microglia, oligodendrocytes, and Schwann cells. The *Drosophila* adult central nervous system (CNS) has five glial subtypes divided into three main categories, namely surface, cortex or neuropil glia.

Surface glia consist of two distinct subtypes, the perineurial and subperineurial glia. Perineurial glia are narrow, oblong cells that make up the outermost covering of the adult CNS (Awasaki et al., 2008). During development, these cells increase their cell division to maintain complete coverage of the adult *Drosophila* nervous system (Avet-Rochex et al., 2012). The function of this glial cell subtype in the adult fly brain remains largely unknown (Edwards and Meinertzhagen, 2010), but a recent study suggests that perineurial glia may be important in the transport of trehalose (the main energy supplying carbohydrate in insects) across the blood-brain barrier (Volkenhoff et al., 2015). Subperineurial glia are large, flat polyploidal cells (Unhavaithaya and Orr-Weaver, 2012) that reside just underneath the perineurial glia layer. Unlike perineurial glia, subperineurial glia undergo endoreplication during larval development to increase their cell size to maintain coverage of the brain through metamorphosis (Unhavaithaya and Orr-Weaver, 2012). These cells contain several tight septate junctions, form the blood-brain barrier of *Drosophila*, and separate the CNS from pathogens, xenobiotics, and the high electrolyte content of the hemolymph ultimately protecting neuronal function (Limmer et al., 2014; Weiler et al., 2017). Consistent with these functions, the transcriptome of surface glia is enriched for gene categories associated with drug metabolism, cell adhesion, and various transporters (DeSalvo et al., 2014).

Cortex glia make contact with the subperineurial glia through adherens junctions and envelope neuronal cell bodies that reside in the cortex providing metabolic support to them (Edwards and Meinertzhagen, 2010). One cortex glial cell can cover many neuronal bodies, which gives these cells a mesh-like appearance (Awasaki et al., 2008).

Located below the cortex are two types of neuropil glia, astrocytes and ensheathing glia. Ensheathing glia have a fibrous lamellar morphology (Awasaki et al., 2008) and act as phagocytes of the brain, similar to mammalian microglia. These glia respond to axonal injury through the *Draper* receptor signaling pathway (Doherty et al., 2009). Astrocyte glial cell bodies are located at the cortex/neuropil border and have projections that are closely associated with neuronal synapses and contain multiple neurotransmitter recycling pathways (Stork et al., 2012). A recent study of the transcriptome of fly astrocytes showed enriched expression of genes involved in metabolism, redox reactions, neurotransmitter synthesis and transport (Ng et al., 2016). RNAi-mediated knockdown of some of these genes revealed alterations in behavior including changes in activity level, activity onset, and mechanical stress induced paralysis (Ng et al., 2016).

It has been established that some glial subtypes express circadian clock genes in a rhythmic manner. In mammals, both astrocytes (Prolo et al., 2005; Marpegan et al., 2011) and microglia (Hayashi et al., 2013; Fonken et al., 2015) rhythmically express Per1 and Per2 proteins. Cultured astrocytes from Per1::luciferase transgenic rats and knock-in mice are capable of maintaining modest rhythms in circadian clock gene expression that can be entrained by physiologically relevant temperature changes (Prolo et al., 2005). Rhythmic expression of several clock genes was also shown in cortical microglia by qRT-PCR (Hayashi et al., 2013; Fonken et al., 2015). Expression of the circadian clock gene *per* in glia have been also suggested in flies (Ewer et al., 1992) and this was confirmed more recently although, it is not clear which glial subtypes express PER protein (Ng et al., 2011).

Although impairments of the circadian system are believed to be involved in accelerated aging, little is known about how the circadian clock in different tissues is altered across the lifespan. In *Drosophila*, PER expression remains robust in central pacemaker neurons (Luo et al., 2012) but is significantly reduced in retinal photoreceptors (Luo et al., 2012; Rakshit et al., 2012). While glia have many important roles in maintaining nervous system homeostasis, it is not known which glial subtypes express the core clock protein PER and whether PER levels remain similar across lifespan or decline with age. To address these questions, we took advantage of the fact that glial subtypes of *Drosophila* have unique expression patterns and can be labeled separately by GFP via cell-type specific drivers. We performed 2-timepoint immunocytochemical experiments to identify *Drosophila* glia subtypes that express PER protein and determined that the PER level in these cells is reduced in old fly brains compared to young.

## Methods

### Fly rearing and genetics

*Drosophila melanogaster* were maintained on diet containing yeast (35 g/l), cornmeal (50 g/l), and molasses (5%). Temperature was maintained at 25 ± 1°C with a 12:12 h light/dark cycle with fluorescent light of luminous energy of 8 ± 2 μmol m^−2^s^−1^. We used mated males in all experiments to minimize differences in lifespan, which may vary with sex and mating status. Males were aged in groups of 50 in polypropylene cages (Genesee Scientific, San Diego, CA) inverted over 35 mm petri dish (BD Falcon, San Jose, CA) containing 15 mL of diet. Diet dishes were replaced every 2–3 days. Young (day 5) and old (day 55) males expressing nuclear GFP in specific glial cell subtypes were obtained by crossing *w;*UAS-GFP with nuclear localization signal (Bloomington Drosophila Stock Center stock 4775) males with females carrying GAL4 drivers expressing in the following glia types: perineurial glia, *NP6293*-GAL4 (Awasaki et al., 2008); subperineurial glia *moody*-GAL4 (Schwabe et al., 2005); cortex glia *NP577*-GAL4 and *NP2222*-GAL4; ensheathing glia *NP6520*-GAL4 (Awasaki et al., 2008) and *mz0709*-GAL4 (Ito et al., 1995); astrocytes *alrm*-GAL4 (Doherty et al., 2009). UAS-GFP with the nuclear localization signal was chosen to clearly discern nuclear overlap between GFP and PER protein; however, some GFP was also visible in the cytoplasm of glial cells.

### Immunocytochemistry (ICC)

Flies for brain dissection were collected at Zeitgeber time (ZT) 22 and ZT10 which correspond to high and low levels of PER protein in wild-type flies, respectively (Long et al., 2014). Whole brain mounts were made using established protocol (Long et al., 2014). Brains were incubated for 48 h in primary antibodies 1:500 chicken monoclonal anti-GFP (Aves Laboratories) and 1:10,000 pre-absorbed rabbit anti-PER, rinsed 6 times in phosphate buffered saline with 0.5% Triton-X (Fisher Scientific, Pittsburgh, PA) and incubated overnight in secondary antibodies Alexa Fluor 488 anti-chicken (1:500) and Alexa Fluor 555 anti-rabbit (1:1,000) (Life Technologies). After the final rinse, brains were mounted on microscope slides in Vectashield mounting media with DAPI (Vector Laboratories, Burlingame, California). Images were taken with a Zeiss LSM 780 NLO scanning confocal microscope (Zeiss) with all laser parameters set for optimal signal in young fly brains at ZT22 for each genotype and then held constant while imaging young ZT10 as well as old ZT22 and ZT10 flies of the same genotype. Young and old *per*^*01*^ mutant flies were dissected and stained along with each genotype using the same protocol. Lack of PER staining signal in *per*^*01*^ mutants was used as a negative control.

### Image analysis and PER quantification

In order to quantify the relative fluorescence of PER signal at ZT10 and ZT22 in each glia subtype, images were reviewed and maximum intensity projections were created using ZEN 2012 software (Zeiss). Due to their location on the outer surface of the adult *Drosophila* brain, in order to capture a sufficient number of surface glial cells for measurement, multiple images of non-overlapping 6 μm stacks were captured in several regions of each brain. The area of focus for perineurial glia was the dorsal brain while the surface of the optic lobes was used for subperineurial glial cells. For all other glial subtypes, a single 11 μm thick stack from each brain was used for PER signal quantification. PER levels were evaluated by measuring the fluorescence intensity in an average of 15 GFP-positive cell nuclei located in the region of interest specified below. After converting the mean level of fluorescence to the Mean Gray Value the intensity was quantified using Fiji ImageJ software (Schindelin et al., 2012). For each stack, measurements of non-specific background fluorescence were taken from the adjacent areas of similar size as glial cell nuclei (avoiding non-specific red speckles). The background values were averaged and subtracted from the averaged PER measurements obtained from that stack. Five to seven brains were used to measure PER at given time point and age. Statistical significance for average intensity of PER staining between young and old brains at ZT22 was calculated by unpaired *t*-test with Welch's correction using GraphPad Prism 6 (GraphPad Prism v6.0;GraphPad Software Inc. San Diego, CA). The *p*- and *t*-values and the degrees of freedom (df) from these measurements are provided in the results section and in the figure captions.

## Results

We investigated which glia subtypes express the circadian clock protein PER in a manner similar to that of the pacemaker neurons and whether the relative amounts of PER signal change with age. To label specific glial cells, we employed the GAL4/UAS system (Brand and Perriman, 1993) using GAL4 lines to drive GFP expression in subtypes of glia with specific location and function in combination with immunocytochemistry to measure PER levels. It has been reported that PER expression in lateral and dorsal pacemaker neurons are equally strong in young and old *Drosophila* brains (Luo et al., 2012); therefore, the presence of PER staining in these neurons was used as a positive control. These cells have rhythmic PER expression with high levels at ZT22 and lack PER at ZT10 in wild type flies (Long et al., 2014); therefore, we selected these time points to examine glial cells.

### PER is expressed in both layers of the surface glia

**Figure 1:**
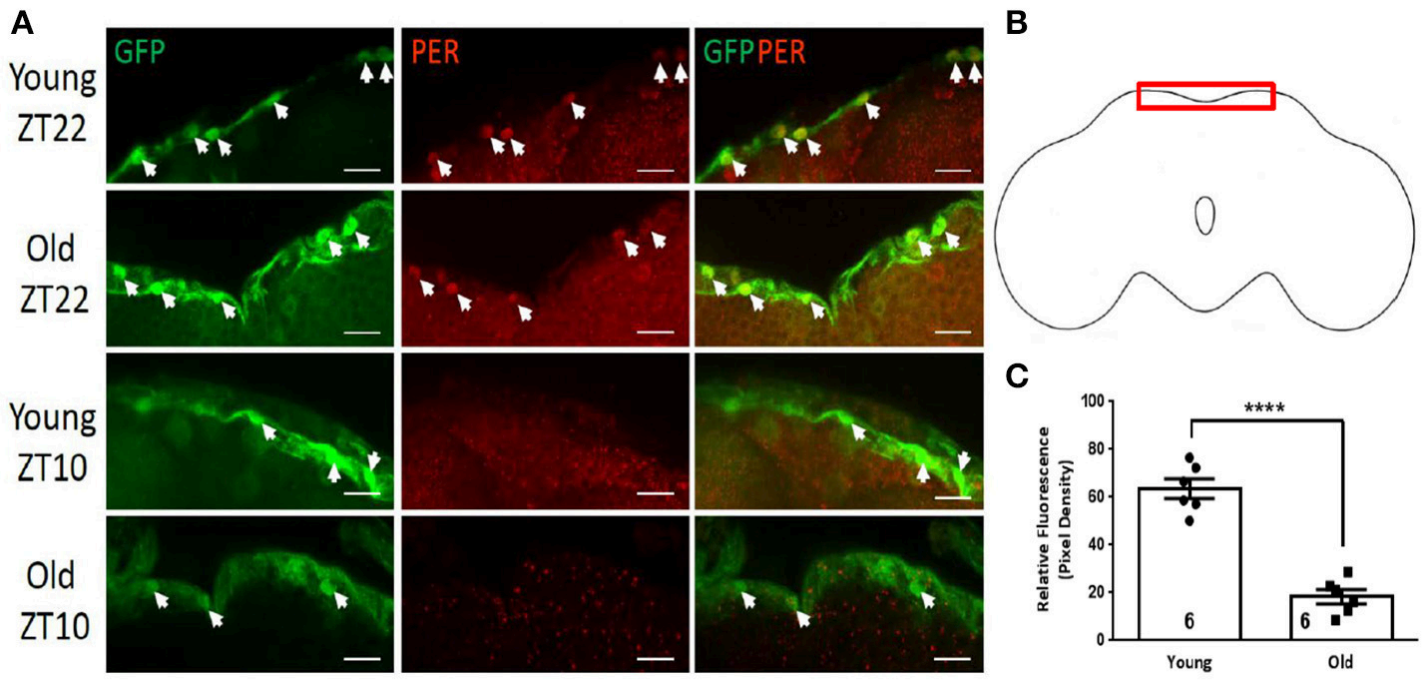
PER expression in perineurial glia of the dorsal brain. GFP-positive perineurial glia covering brain surface visualized in *NP6293-*GAL4>GFP flies. **(A)** Representative brains showing GFP, PER, and combined labeling of the cell nuclei (arrowheads) in young (5 days) and old (55 days) brains at ZT22. At ZT10, PER is absent in perineurial glia from both young old brains. Scale bars equal 10 μm. **(B)** Brain outline indicating location of cells shown in **(A)** in the dorsal surface region. **(C)** Graph showing the average relative fluorescence of PER in perineurial glia. PER levels at ZT22 were significantly lower in old brains (^****^p ≤ 0.0001, *t* = 8.928, df = 9.171). Number of brains analyzed are shown within each bar; error bars indicate standard error of the mean (SEM).

**Figure 2:**
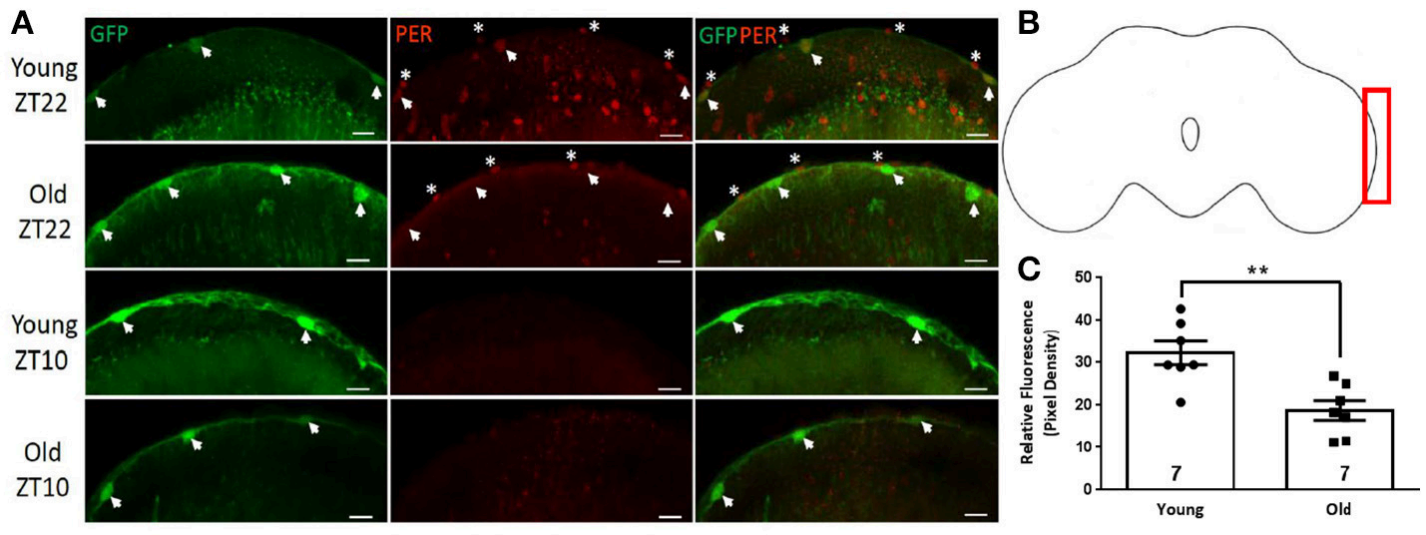
Subperineurial glial cells express PER. Close up of the optic lobe surface in brains of *moody-*GAL4>GFP flies. **(A)** Large GFP-positive nuclei of subperineurial glia (arrowheads) are PER-positive in the brains of young (5 day) flies at ZT22, but PER protein is almost absent in glia of old brains at ZT22, although PER is detected in the outer layer of perineurial glia not labeled by GFP (asterisks). Other PER-positive cells in young brains at ZT22 not marked by GFP are likely different types of glia. Subperineurial glia in young and old brains are PER-negative at ZT10. Scale bars equal 10 μm. **(B)** Brain outline indicating location of cells shown in **(A)** at the surface of the optic lobe. **(C)** Graph showing the average relative fluorescence of PER in subperineurial glia at ZT22. PER level is significantly lower in old brains (^**^p = 0.0031, *t* = 3.719, df = 11.56). Number of brains analyzed are shown within each bar; error bars indicate SEM.

Surface glia consist of two distinct glial subtypes namely perineurial and subperineurial glia. Perineurial glial cells were labeled by crossing UAS-GFP to *NP6293*-GAL4 driver line, which specifically marks this layer of glia (Awasaki et al., 2008). All GFP-positive nuclei of cells on the dorsal surface of the brain showed PER signal at ZT22 but not at ZT10, suggesting rhythmic expression in the perineurial glia in young brains (Figures 1A,B). PER expression persisted in the brains of old flies but the average signal at ZT22 was significantly reduced (Figure 1C, *p*-value < 0.0001, *t*-value = 8.928, df = 9.171). Subperineurial glial cells were visualized via *moody*-GAL4 driver line (Schwabe et al., 2005) combined with UAS-GFP. We observed that GFP-labeled cells surrounded the entire brain in both young and old flies. PER signal was quantified in GFP positive subperineurial glia surrounding the optic lobe (Figures 2A,B). Although some GFP leaked and was observed in the cytoplasm of the subperineurial glia predominant signal came from their large nuclei. Subperineurial glia showed nuclear PER signal at ZT22 (Figure 2A, arrowheads) but not at ZT10 in brains of young flies suggesting rhythmic expression of this protein. PER was discernible from the background in old brains at ZT22 but the average PER signal was significantly reduced compared to young flies (Figure 2C, *p*-value ≤ 0.0031, *t*-value = 3.719, df = 11.56).

### PER is weakly expressed in cortex glia at ZT22

**Figure 3:**
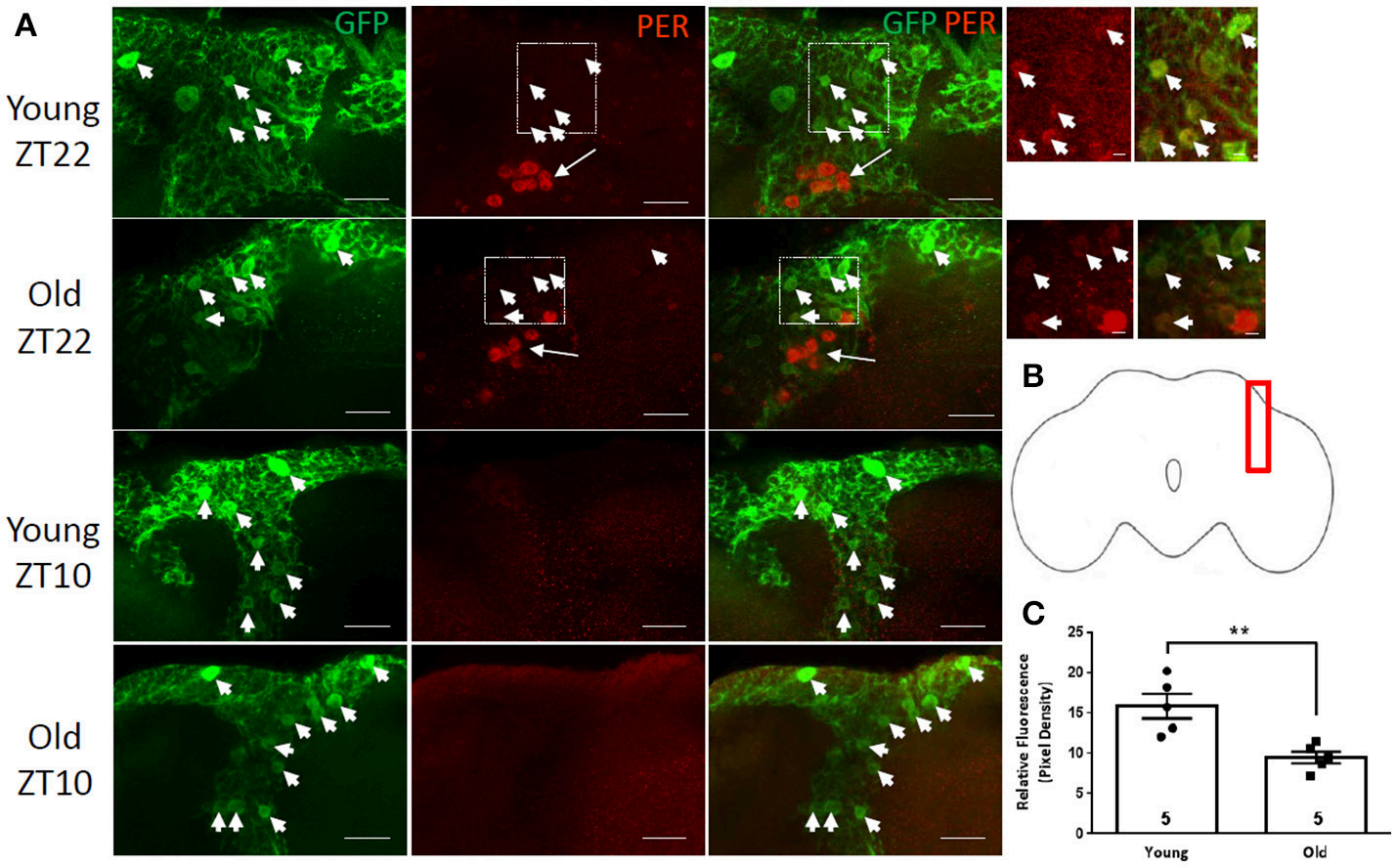
PER is weakly express in GFP-positive cortex glia. Images of the region containing PER-positive dorsal lateral neurons (arrows) and GFP-positive cortex glia (arrowheads) in brains of *NP2222*-GAL4>GFP flies. **(A)** In comparison to neurons, very weak PER staining is observed in GFP-positive cortex glia at ZT22 in young flies and is further reduced in old flies. No PER is detected in young or old brains at ZT10. Scale bars equal 10 μm. Right: enlarged images of the outlined regions show weak but discernible PER signal in cortex glia in young and old brains at ZT22. Scale bars equal 2 μm. **(B)** Brain outline indicating location of cells shown in **(A)** in the lateral region of the brain. **(C)** Graph showing the average relative PER fluorescence in cortex glia at ZT22. PER level is significantly lower but still detectable in old brains (^**^p = 0.0099, *t* = 3.784, df = 5.743). Number of brains analyzed are shown within each bar; error bars indicate SEM.

Cortex glia surround neuronal cell bodies that reside underneath the surface glia. To visualize cortex glia cells, we used two drivers, *NP2222*-GAL4 or *NP577*-GAL4 (Awasaki et al., 2008) combined with UAS-GFP. GFP-positive cells were abundant in both young and old flies having a mesh-like appearance as described previously (Awasaki et al., 2008). Given their large population and prolific distribution in the cortex, we focused on a small subset of GFP-positive cortex glia in the vicinity of the dorsal lateral pacemaker neurons (Figures 3A,B). PER signal was detected in both cortex glia lines at ZT22 but not at ZT10 similar to the circadian expression of PER in the lateral neurons (Figure 3A). The relative level of PER signal in cortex glia in brains of *NP2222*-GAL4>GFP flies was much lower than in the lateral neurons located nearby, but was present at ZT22 and not at ZT10 (Figure 3A). Analysis of PER signal in cortex glia in this region showed that the average PER levels were significantly reduced in old fly brains compared to young (Figure 3C, *p*-value ≤ 0.0099, *t*-value = 3.784, df = 5.743).

### Variable PER expression in the neuropil glia

**Figure 4:**
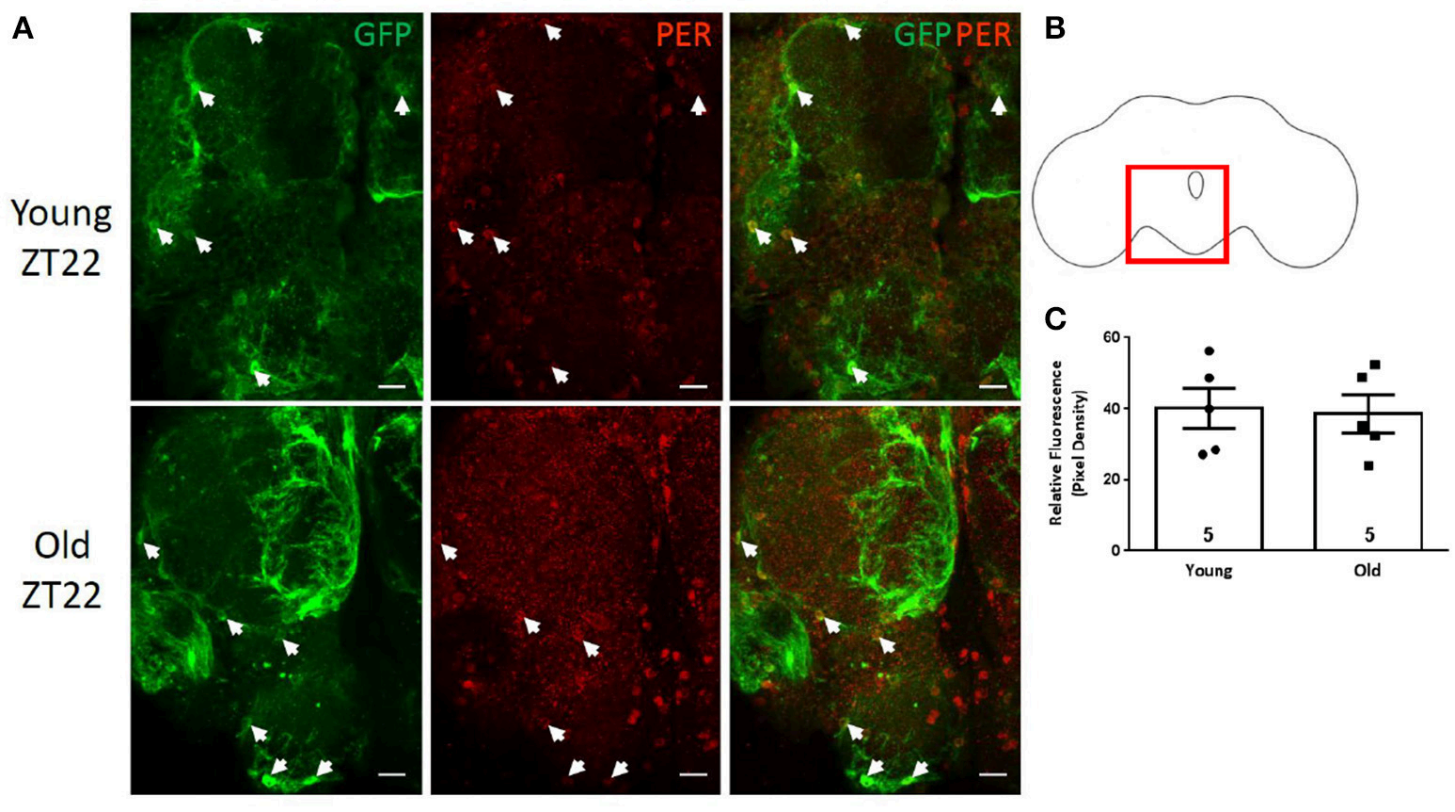
PER is variably expressed in the neuropil ensheathing glia. Ensheathing glia are labeled with GFP (arrowheads) in *NP6520-*GAL4>GFP brains. **(A)** PER signal is detected in a subset of GFP-positive cells at ZT22 in the central brain of 5 and 55 day old flies. PER levels vary from cell to cell. No PER was detected in young or old brains at ZT10 (data not shown). Scale bars equal 10 μm. **(B)** Brain outline indicating location of cells shown in **(A)** in the region of the ventral central brain. **(C)** Graph showing the average relative PER fluorescence in ensheathing glia. PER levels are not significantly different between young and old flies (*p* = 0.8452, *t* = 0.2017, df = 7.961). Number of brains analyzed are shown within each bar; error bars indicate SEM.

Neuropil glia consists of two morphologically distinct subtypes, the ensheathing glia and astrocytes. Ensheathing glia were visualized via *mz0709*-GAL4 (Ito et al., 1995) or *NP6520*-GAL4 (Awasaki et al., 2008) drivers combined with UAS-*GFP*. PER was detected in GFP-positive cells in both lines; however, *mz0709*-GAL4 has been reported to drive expression also in the subperineurial glia (Dutta et al., 2016); therefore, *NP6520*-GAL4>GFP flies were used for PER signal measurement. GFP-positive cells were observed at the border between cortex and several neuropil compartments in the central brain (Figures 4A,B). At ZT10, these ensheathing glial cells were negative for PER signal in both young and old brains (not shown); however, many of these cells were PER positive at ZT22 in both ages (Figure 4A). The intensity of PER signal was variable from brain to brain and cell to cell and there was no significant difference in average PER signal between young and old brains (Figure 4C, *p*-value = 0.8452, *t*-value = 0.2017, df = 7.961).

**Figure 5:**
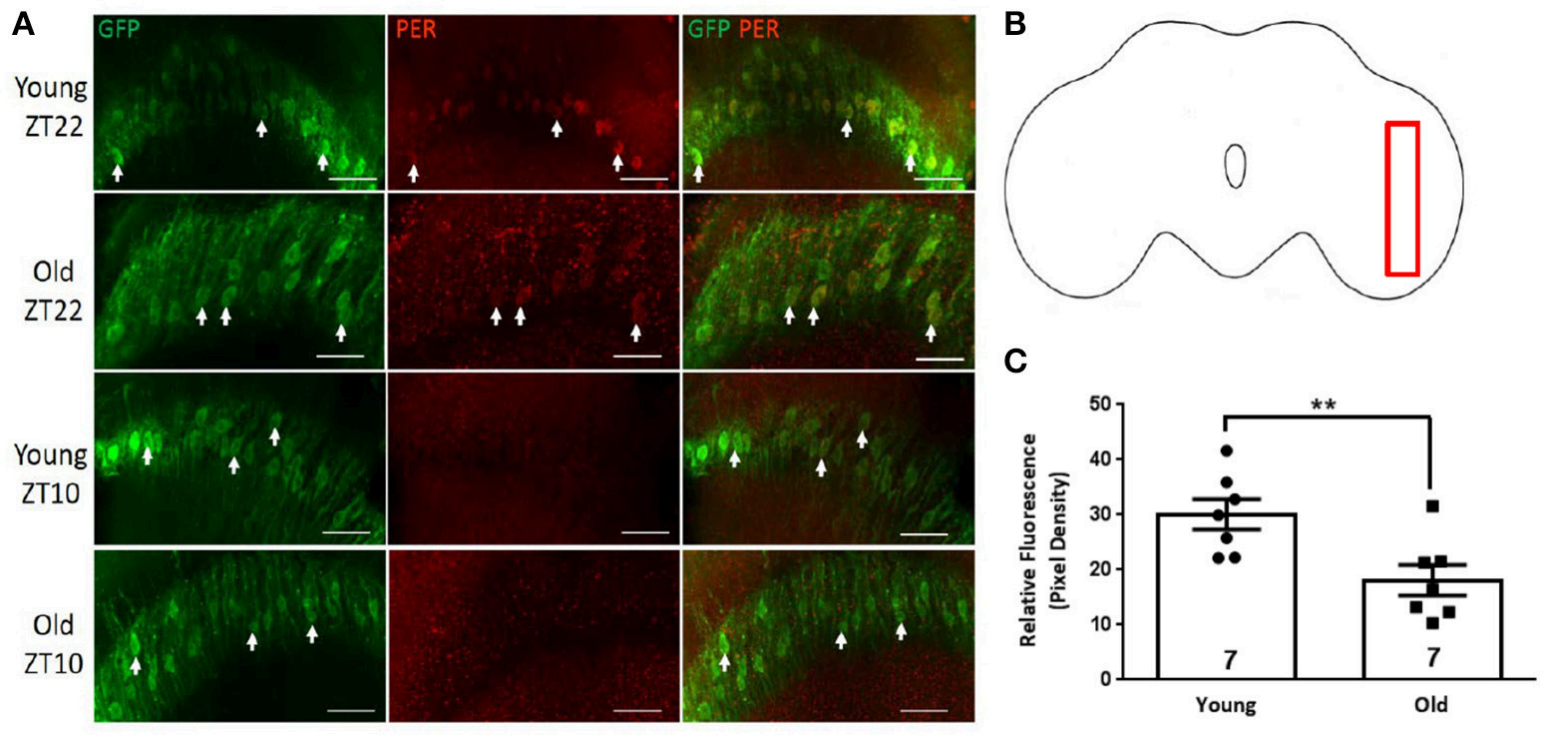
PER expression in glia of the medulla. A prominent group of GFP-positive giant glial cells in the medulla of *NP6520*-GAL4>GFP brains. **(A)** PER protein was detected in young brains and at a lower level in old brains at ZT22 but was absent in both at ZT10. Scale bars equal 20 μm. **(B)** Brain outline indicating location of cells shown in **(A)** in the region of the medulla. **(C)** Graph showing average relative PER fluorescence of in giant glia at ZT22. PER level is significantly lower in old brains (^**^p = 0.0100, *t* = 3.056, df = 12). Number of brains analyzed are shown within each bar; error bars indicate SEM. Arrowheads indicate GFP labeled glial cell nuclei.

Another group of glial cells marked with GFP via *NP6520*-GAL4 driver was observed in the medulla segment of the optic lobe (Figures 5A,B). Based on their position and large oblong nuclei, these cells appear to represent the giant glial cells of the medulla (Tix et al., 1997). Cell nuclei were PER negative at ZT10, but PER was detected at ZT22 albeit with somewhat variable intensity from cell to cell (Figure 5A). Nevertheless, the average intensity of PER signal was significantly reduced in these cells in the brains of old files compared to young (Figure 5C, *p*-value ≤ 0.0100, *t*-value = 3.056, df = 12) indicating that PER levels in these glial cells are reduced as the function of age similar to other glia types discussed above.

**Figure 6:**
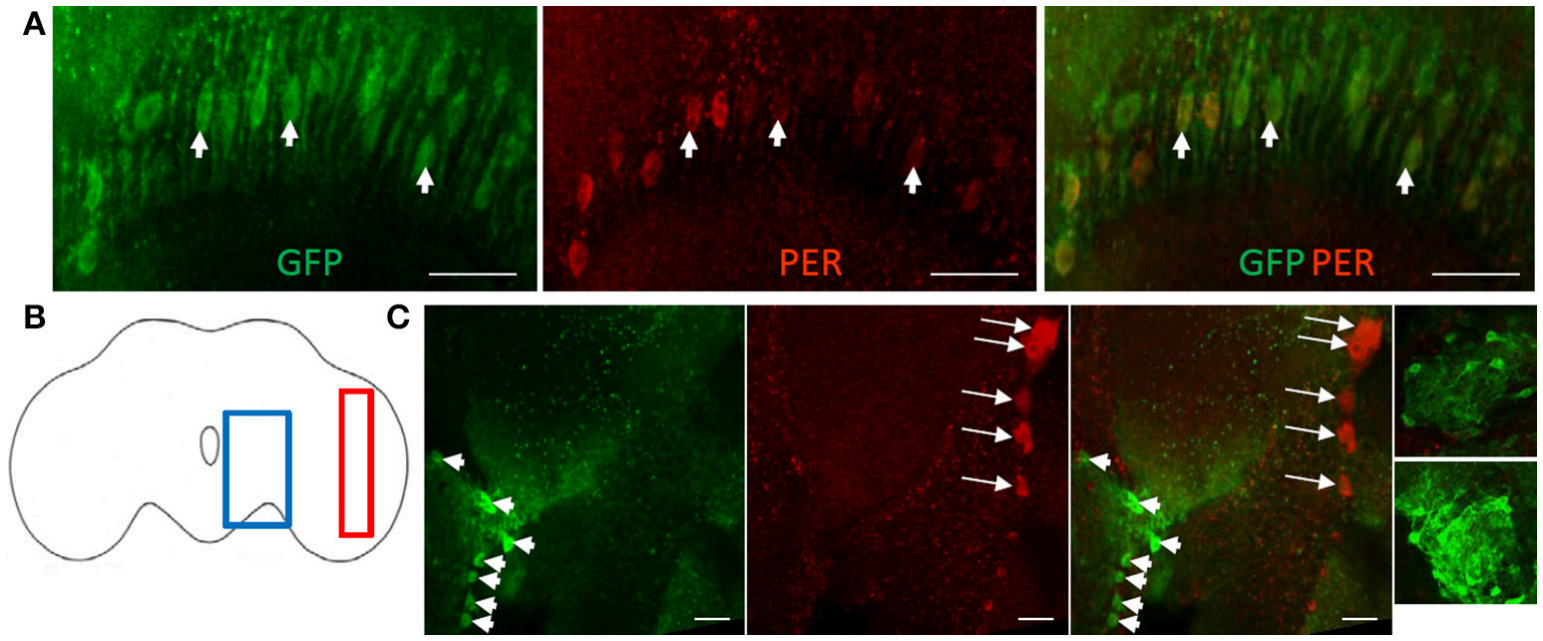
Astrocyte-like cells labeled with GFP via *alrm-*GAL4 driver. **(A)** This driver appears to be active in the same PER-positive giant glial cells of the medulla that were marked with ensheathing glia driver (see Figure 5). Scale bars equal 20 μm. **(B)** Brain outline indicating approximate location of cells shown in **(A)** in red and cells shown in **(C)** in blue. **(C)** Astrocytes labeled with GFP via *alrm-*GAL4 and located in the central brain (arrowheads) are PER-negative while ventral lateral neurons located nearby (arrows) show PER signal at ZT22. Scale bars equal 10 μm. Two small images on the right show additional PER-negative astrocytes in the central brain.

A second group of prominent neuropil glia are astrocytes which were visualized via *alrm*-GAL4 driver (Doherty et al., 2009) combined with UAS-GFP. Interestingly, it appears that *alrm*-GAL4 marked as GFP-positive the same giant glial cells in the medulla that were also labeled via ensheathing glia *NP6520*-GAL4 driver. PER was again detected in these cells at ZT22 (Figures 6A,B) with the average signal lower in old flies (not shown). This suggests that these cells share features of both ensheathing and astrocyte glial cells.

In mammals, astrocytes located among the central clock neurons in the SCN show robust oscillations in Per-reporter. Therefore, we investigated whether astrocytes located in the central brain neuropil are PER-positive in flies. These cells were marked with GFP via *alrm*-GAL4 driver and due to the leakiness of nuclear GFP also show some projections that extended into the neuropil (Figure 6C). GFP-positive astrocytes were examined in several areas of the central brain but PER protein staining was not detected in any of these cells at either ZT22 or ZT10 while nearby ventral lateral pacemaker neurons were PER-positive at ZT22 as expected (Figures 6B,C).

## Discussion

In this study, we show that most glial cell subtypes of the adult *Drosophila* CNS express PER in a manner suggesting that circadian clock may function in these cells. These glial subtypes include perineurial glia, subperineurial glia, cortex glia, ensheathing glia of the central brain and the giant glia located in the medulla. While rhythmic PER expression in the medulla was reported previously (Suh and Jackson, 2007; Gorska-Andrzejak et al., 2009), our data suggest clock function in several other types of glial cells. However, the astrocyte glia appear to be an exception as we did not detect PER protein in these cells at either time point examined.

Our finding that perineurial and subperineurial glia express PER protein is consistent with a recent study of the surface glia transcriptome of adult *Drosophila* compared to the transcriptomes of all neurons, all glia, and to total brain lysates (DeSalvo et al., 2014). While not the focus of the study, their data do list *per* mRNA and other circadian clock genes as expressed in the surface glia (DeSalvo et al., 2014). Moreover, the core clock gene *Clk* was identified as one of the top 50 genes enriched in surface glia when compared to all glia (DeSalvo et al., 2014). The perineurial and subperineurial glia have distinct non-overlapping roles in the formation and maintenance of the blood-brain barrier that are not well understood (Awasaki et al., 2008; DeSalvo et al., 2014); the presence of the circadian clock in these cells may help to understand their functioning in the future.

Cortex glia constitute about 20% of the glia in the adult *Drosophila* brain (Kremer et al., 2017), but this subtype is relatively understudied in flies. Based on our results, cortex glia express PER protein similarly to the pacemaker neurons albeit at a much lower level even in young flies. However, we cannot exclude that cortex glia in other brain regions could show higher PER levels. Cortex glia are presumed to provide trophic support to the neuronal cell bodies they envelop (Edwards and Meinertzhagen, 2010). A recent study supports this idea by demonstrating that genes involved with β-oxidation are expressed in cortex glia suggesting that these cells may generate and transport ketone bodies (Schulz et al., 2015). Cortex glia are known to produce Ca^+2^ oscillations and disruptions of these oscillations by mutations in the glial-specific Na^+^/Ca^2+^, K^+^ exchanger encoded by *zydeco* significantly decrease seizure threshold in flies (Melom and Littleton, 2013). While cortex glia are important for neuronal health and function, the role of potentially low amplitude PER oscillations in these cells has yet to be addressed.

Ensheathing glial cells are closely associated with neuronal arborizations and synaptic regions. We determined that these cells express PER in the central brain in *Drosophila*. It has been reported that the equivalent mammalian cells, the microglia express Per1 and Per2 in a circadian manner (Hayashi et al., 2013; Fonken et al., 2015). The ensheathing glia of *Drosophila* uniquely express key components of the glial phagocytic machinery such as the engulfment receptor *Draper* (Doherty et al., 2009). Interestingly, a recent study comparing circadian transcriptome in heads of young and old flies indicated that *drpr* mRNA show a rhythmic profile in young flies but the rhythm is dampened in old (Kuintzle et al., 2017).

We determined that the astrocyte glia in the central brain in the vicinity of the pacemaker neurons do not express PER. These results are consistent with a previous report that the astrocytes of the central brain are PER/TIM negative (Suh and Jackson, 2007). The absence of PER in fly astrocytes is somewhat unexpected given that mammalian astrocyte cultures from Per1::luciferase transgenic rats and knock-in mice are capable of maintaining modest rhythms in circadian clock genes expression (Prolo et al., 2005; Marpegan et al., 2011). In fact, several recent studies have demonstrated that mammalian astrocytes are important for controlling circadian timekeeping. Astrocyte-specific loss of core clock gene BMAL1 (the mammalian ortholog of *cycle*) via two independent methods was shown to alter circadian locomotor activity whereas expression of clock-associated kinase CK1ε in astrocytes was sufficient to lengthen the period of PER oscillations (Tso et al., 2017). Other recent studies also demonstrated astrocytes roles in circadian timekeeping through glia-neuron communication involving different signaling molecules (Barca-Mayo et al., 2017; Brancaccio et al., 2017). Consistent with the lack of PER in the fly central brain astrocytes, a study of the astrocyte transcriptome did not identify any of the core circadian clock genes (Ng et al., 2016). While astrocytes in both flies and mammals associate with neuronal synapses and have similar star-shaped morphologies and molecular markers, it is conceivable that circadian clock function could have been acquired later in evolution aided by the substantial proliferation of glia in mammals (Kremer et al., 2017).

Little is known about clock-controlled output processes in *Drosophila* glia. In the lamina of the fly visual system, glial cells show rhythmic changes in volume coordinated with the volume changes in the photoreceptor-contacting interneurons (Gorska-Andrzejak, 2013). These rhythmic changes in structure coincide with the rhythmic expression of the α-subunit of the sodium pump, Na^+^/K^+^-ATPase, which is in high abundance in glia and its rhythmic expression is *per*-dependent, as *per*^*01*^ mutants lack rhythmic expression of this subunit (Gorska-Andrzejak et al., 2009). A recent study implicates glial cell oscillators in the control of *Gclc*, a rhythmically expressed component of the rate-limiting enzyme in glutathione synthesis (Chow et al., 2016). This study reported that pan glial knockdown of the circadian clock gene *cycle* via *loco-*GAL4>*cycRNAi* was sufficient to significantly decrease rhythmicity of *Gclc* expression (Chow et al., 2016).

The ubiquitous and rhythmic expression of PER in glia reported here opens more questions regarding the functional significance of glial clocks. The role of glia in locomotor activity rhythms is not clear. Pan-glial knockdown of the circadian clock genes *per* or *cryptochrome* via RNAi failed to alter the free-running behavior of adult *Drosophila* (Ng et al., 2011). Yet, other studies have shown that *Drosophila* glial cell functions are required for behavioral rhythmicity (Jackson, 2011; Ng et al., 2011, 2016; Jackson et al., 2015). Finally, pan-glia knockdown of several astrocyte enriched genes can cause significant changes in activity level, sensitivity to mechanical stress, and/or alterations in circadian locomotor activity (Ng et al., 2016).

One of the important findings of our study is that, with the exception of the central brain neuropil glia, all PER expressing glial subtypes display significant age-related decline in PER protein levels. These data are consistent with the age-related decrease in PER protein reported by Western blot in whole heads (Luo et al., 2012; Rakshit et al., 2012; Kuintzle et al., 2017). Previous immunofluorescent studies detected PER decline in the retinal photoreceptors of old flies (Luo et al., 2012; Rakshit et al., 2012). Our data now show that similar decline occurs in the majority of glial cells. Although the reasons for age-related decrease in PER protein shown here are not known, it could be related to reported reduction of TIM protein in the heads of old flies (Luo et al., 2012; Rakshit et al., 2012). TIM protein is known to be required for PER stability (Hardin, 2011). It is also well known that PER is an essential repressor of CLK/CYC-activated circadian transcription of target genes (Hardin, 2011); therefore, our data suggest that the repressive arm of the circadian clock weakens in glia during aging due to decline of nuclear localized PER protein. Consistent with this hypothesis, a recent RNA-seq study showed that *per* mRNA expression is higher in the heads of old flies compared to young while protein levels are decreased in old (Kuintzle et al., 2017).

Our detailed analysis of PER expression suggests that circadian clock may function in several glial subtypes. These data should facilitate future functional analysis of glial circadian clocks and their roles in homeostasis of the nervous system. The age-related decline in PER protein expression in various glial subtypes may provide new ways of investigating the physiological processes that decline with age.

## Author contributions

This study was conceived, analyzed, and written by DL and JG. All experiments were performed by DL.

### Conflict of interest statement

The authors declare that the research was conducted in the absence of any commercial or financial relationships that could be construed as a potential conflict of interest.
